# Clinical features and overall survival among elderly cancer patients in a tertiary cancer center

**DOI:** 10.1590/S1679-45082015AO3067

**Published:** 2015

**Authors:** Yuri Philippe Pimentel Vieira Antunes, Diogo Diniz Gomes Bugano, Auro del Giglio, Rafael Aliosha Kaliks, Theodora Karnakis, Lucíola de Barros Pontes

**Affiliations:** 1 Hospital Israelita Albert Einstein, São Paulo, SP, Brazil.; 2 Hospital do Coração, São Paulo, SP, Brazil.

**Keywords:** Neoplasms/epidemiology, Neoplasms/mortality, Medical oncology, Aged

## Abstract

**Objective:**

To evaluate the epidemiological profile and overall survival of a large population of elderly individuals diagnosed with solid tumors in a tertiary hospital.

**Methods:**

This retrospective study included patients aged >65 years, diagnosed with solid tumors between January 2007 and December 2011, at *Hospital Israelita Albert Einstein*, São Paulo, Brazil. The medical records were reviewed to obtain information about clinical variables and overall survival.

**Results:**

A total of 806 patients were identified, and 58.4% were male. Mean age was 74 years (65 to 99 years). The most common types were prostate (22%), colorectal (21%), breast (19%), and lung cancer (13%), followed by bladder (8%), pancreas (6%), and other types (11%). The majority of patients were diagnosed at early stage disease. After a median follow-up of 27 months (15 to 45 months), 29% of the patients (234/806) died, predominantly in the group older than 70 years. For the entire cohort, the median 2-year survival rate was 71%. Median overall survival was not reached within the study period. In a multivariate analysis, age (HR: 1.35; 95%CI: 1.25-1.45; p<0.001) and disease stage (HR: 1.93; 95%CI: 1.75-2.14; p<0.001) were independent negative predictors of poor survival.

**Conclusion:**

The most prevalent tumors were prostate, colorectal, breast, and lung cancer, with the larger proportion diagnosed at initial stages, reflecting the great number of patients alive at last follow-up.

## INTRODUCTION

Cancer is a leading cause of death in the elderly population. Over 50% of all types of cancer and approximately 70% of cancer-related deaths occurring in individuals aged 65 years or older.^1^ Currently in Brazil, there are more than 20 million individuals over the age of 65; official data estimate that this number will be close to 39 million in 2040.^2,3^

Elderly patients are a heterogeneous population with regard to comorbidities, which may interfere with treatment and prognosis. In addition, there are differences in the prevalence of specific types of cancer and risk factors in the elderly as compared to younger individuals. Thus, the first step in a reasonable approach to screening and healthcare policies for the elderly is to determine the epidemiological features of cancer in this age group for each country or region.^4,5^

## OBJECTIVE

To evaluate the epidemiological profile and overall survival of a large population of elderly individuals diagnosed with solid tumors in a tertiary hospital.

## METHODS

We performed a cross sectional cohort study using the registry of *Hospital Israelita Albert Einstein* as our database. This is a private hospital located in the city of São Paulo (SP), Brazil. Patients aged 65 or older who were diagnosed with solid tumors, and registered between January 2007 and December 2011, were the subjects of this analysis.

The charts and electronic institutional databases were reviewed to obtain information about sex, age at diagnosis, type of cancer, stage at diagnosis, and overall survival. We were not able to establish cancer-specific and all-cause mortality for a large proportion of patients.

Overall survival was defined as duration of time from diagnosis until death and patients were censored if they were lost to follow-up (collected until March 21, 2013). Overall survival was estimated using Kaplan-Meier methods. The impact of each treatment on survival was calculated using a Cox regression model.

TNM Classification of Malignant Tumors, seventh edition, was used to describe and categorize all different cancer stages in this article.

This project was approved by the Institutional Ethics Committee, CAAE: 19159813.3.0000.0071. All analyses were performed on Stata SE 10.1 (StataCorp. College Station, Texas, United States) with a two-sided alpha-value of 0.05, unless otherwise stated.

## RESULTS

### Clinical and demographic profile

Between 1^st^ January 2007 and 31^st^ December 2011, 806 patients were identified, 58.4% of whom were male. Mean age was 74 years (65 to 99 years), distributed by age groups as follows: 65 to 69 years (32%); 70 to 79 years (45%); ≥80 years (23%).

The most common types of cancer were prostate (22%), colorectal (21%), breast (19%), and lung cancer (13%), followed by bladder (8%), pancreas (6%), and other types (11%). Less common cancers included central nervous system, thyroid, duodenal, gastric, and esophageal cancer, and the majority of patients were diagnosed with early stage disease (Table 1). The only exception was in those with lung cancer, in whom 43% of cases were diagnosed with advanced disease.

**Table 1:** Distribution by stage of the most prevalent cancer types in 806 elderly patients diagnosed with solid tumors at *Hospital Israelita Albert Einstein* (2007–2011)

Cancer type	n (%)	Cancer stage n (%)					Not reported n (%)
		0	1	2	3	4	
Prostate	175 (100)	-	4 (2.3)	137 (78.3)	21 (12)	13 (7.4)	-
Colorectal	171 (100)	9 (5.2)	46 (26.9)	51 (29.9)	38 (22.2)	25 (14.6)	2 (1.2)
Breast	152 (100)	19 (12)	65 (43)	39 (25.8)	22 (14.6)	7 (4.6)	-
Lung	106 (100)	-	28 (26.4)	10 (9.4)	21 (19.8)	46 (43.4)	1 (1)
Bladder	67 (100)	33 (49)	18 (27)	5 (8)	-	11 (16)	-
Pancreas	46 (100)	4 (8.7)	5 (10.9)	13 (28.2)	3 (6.5)	20 (43.5)	1 (2.2)
Gastric	34 (100)	-	5 (14.7)	4 (11.8)	7 (20.6)	14 (41.1)	4 (11.8)
Thyroid	22 (100)	-	10 (46)	2 (9)	6 (27)	4 (18)	-

*The description of cancer stage for the less frequent cancers is not shown here. Central nervous system tumors are not staged using the TNM classification.

### Overall survival

**Figure 1:**
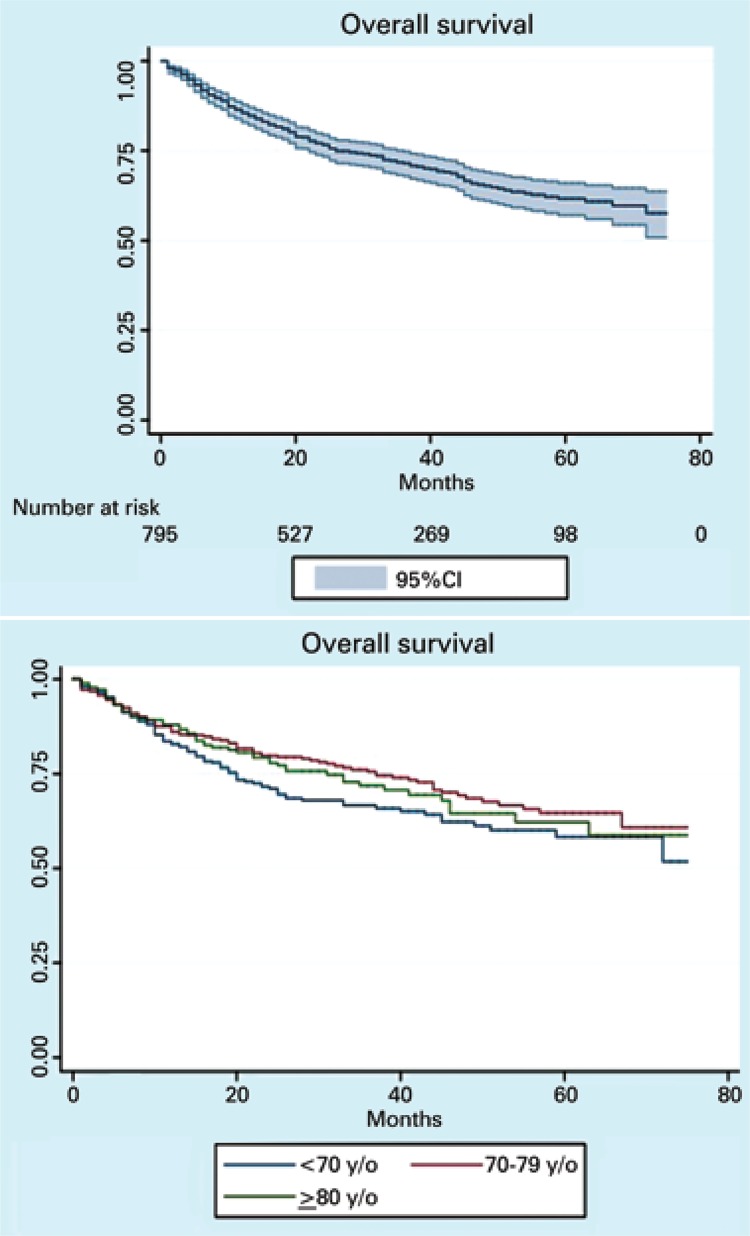
Age-specific overall survival in elderly patients diagnosed with solid at *Hospital Israelita Albert Einstein*, São Paulo, Brazil (2007-2011)

**Figure 2:**
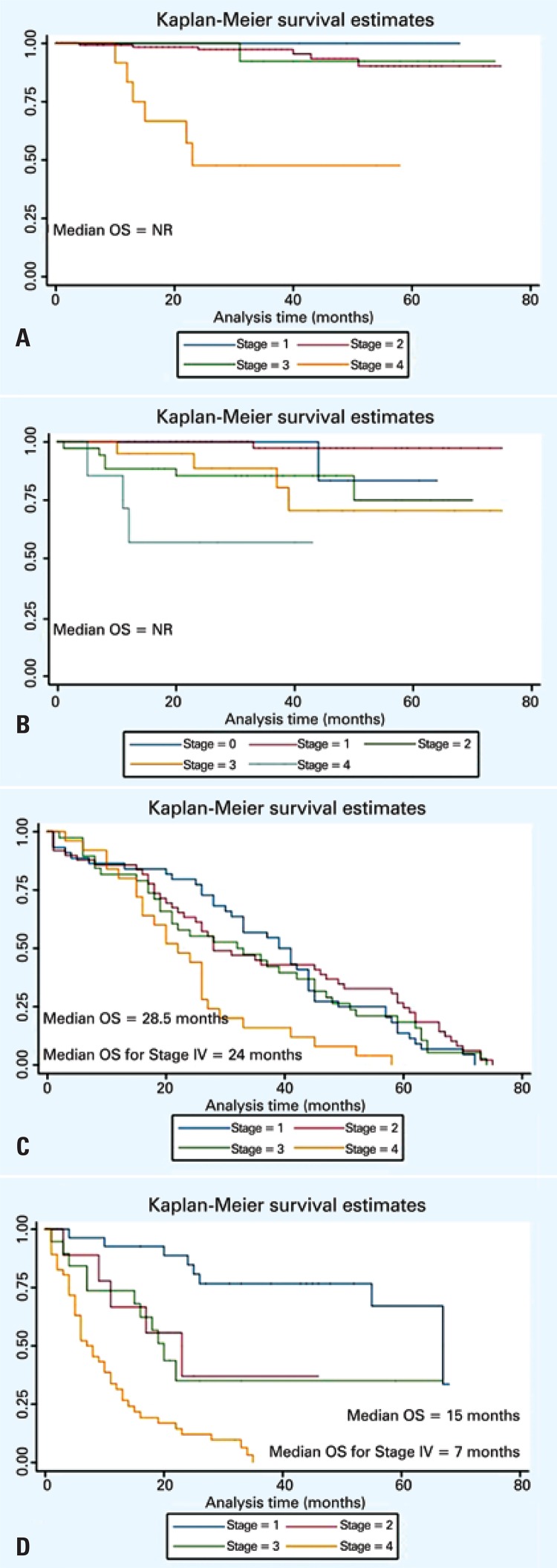
Overall survival for patients diagnosed with prostate (A), breast (B), colorectal (C) and lung cancer (D) according to stage in elderly patients diagnosed with solid tumors at *Hospital Israelita Albert Einstein*, São Paulo, Brazil (2007-2011)

After a median follow-up of 27 months (15 to 45 months), 29% of patients (234/806) died, predominantly in the group with age older than 70 years (Table 2). For the entire cohort, the median 2-year survival rate was 71% (Figure 1). Survival rates were worse among lung cancer patients (Figure 2). Median overall survival was not reached within the study period.

**Table 2:** Distribution by age group and cancer type in 234 elderly patient deaths in a cancer center in São Paulo, Brazil

	n (%)
Deaths	
Yes	234 (29.1)
No	572 (70.9)
Age distribution of patients who died (years)	234
65-69	54 (23)
70-79	88 (37.6)
≥80	92 (39.3)
Cancer types	234
Lung	69 (29.5)
Colorectal	49 (21)
Pancreatic	30 (12.8)
Bladder	24 (10.2)
Central nervous system	16 (6.8)
Breast	15 (6.4)
Prostate	13 (5.5)
Others (gastric, esophageal, and thyroid cancer)*	18 (7.7)

* Gastric, esophageal and thyroid cancer.

For patients with breast and prostate cancer, due to short median follow-up, no mature data on overall survival are currently available; however, the 2-year survival rate was 90 and 92.6%, respectively. Regarding patients with colorectal and lung cancer, median overall survival was 28.5 months and 15 months for all stages, respectively. Considering only patients with stage IV disease, the median overall survival was 24 months for colorectal, and 7 months for lung cancer. In a multivariate analysis using a model including cancer type, age, and disease stage at initial diagnosis, age (HR: 1.35; 95% confidence interval − 95%CI: 1.25-1.45; p<0.001) and disease stage (HR: 1.93; 95%CI: 1.75-2.14; p<0.001) were independent negative predictors of poor survival.

## DISCUSSION

In this study we aimed to evaluate the epidemiological profile of a population of elderly patients diagnosed with solid tumors, in a private hospital in Brazil. Our data showed a slight predominance of males (58%). The most prevalent cancers were prostate, breast, colon, and lung. Such patterns are consistent with recent data from the *Instituto Nacional de Câncer José Alencar Gomes da Silva* (INCA) and the American Cancer Society (ACS),^6,7^ which include these tumors within the five most common for all age groups, indicating the representativeness of our sample. The only exceptions were the lower incidence of cervical and stomach tumors as compared to statistics for the general population,^6,8^ most likely due to selection bias of our population.

Our results also showed that tumors were diagnosed at earlier stages than the national average,^9^ probably again as a result of the characteristics of a private medical center. Presumably, a large proportion of these patients were properly followed by their family physicians and might have undergone screening at a higher frequency than the general population.^10,11^ Besides, our findings regarding staging were in agreement with those reported by Surveillance*, *Epidemiology, and End Results (SEER) statistics from 2000 to 2010,^12^ in which a large proportion of localized tumors were diagnosed as colon, prostate, and breast cancer, in individuals older than 65 years. On the other hand, for lung cancer, SEER data showed 50.6% of cases as stage IV disease, comparable to 43.4% among our patients.^12^

Early detection of cancer can result in less aggressive treatment and better outcomes.^7^ Currently, there are many controversies about the use of cancer screening tests in older people, as indicated by different age cutoffs recommended by diverse guidelines, mainly due to the low number of clinical trials in this subgroup.^13,14^ To help guide individual decisions regarding cancer screening in this heterogeneous population, considering potential risks and benefits and taking into account life expectancy, it would be important to perform a geriatric assessment of each patient before making any screening decisions.

In Brazil, 66% of all cancer-related deaths between 2007 and 2011 occurred in patients older than 60 years.^15,16^ In our sample, lung (30%) and colon (21%) cancers had the highest death rates. This pattern diverges from national trends, in which breast and prostate are the main culprits for cancer mortality.^15,16^ Moreover, our data show more similarity with the North American population, in which the number of deaths estimated for 2013 for lung and colon cancer lead the rankings with 159,480 and 50,830 deaths, respectively.^7^

To what extent the trends in mortality of our patient population are representative of the elderly in Brazil remains to be determined. Of note, two factors were significantly associated with worse outcomes, as revealed by our multivariate analysis: age (HR: 1.35; 95%CI: 1.25-1.45) and clinical stage at diagnosis (HR: 1.93; 95%CI: 1.75-2.14). Specifically, patients older than 80 years accounted for 39% of all deaths in our cohort; it was not possible, however, to determine whether these deaths were cancer-related or due to other causes. Indeed, there is evidence showing that relative survival by age at diagnosis in patients with all cancer types, combined at the 2-year follow-up, is higher in patients aged under 65 years than in those aged over 65 years (75.1% *versus* 69.7%).^12^

Lung cancer is the leading cause of death in developed countries.^17^ In the United States, the median age at diagnosis is 71 years, with 68% of individuals diagnosed at 65 years or older.^17^ Similar epidemiological data are found in colon cancer, in which approximately 70% of patients are diagnosed at age >65 years. According to SEER, 21.7% of lung cancer patients older than 65 years are alive at the 2-year follow-up,^12^ which does not differ from the individuals with lung cancer younger than 65 years (26.5%).^12^ In regard to colon cancer, our data were also comparable to SEER’s, in which 73% of patients older than 65 years were alive at the 2-year follow-up, *versus *79.7% for those younger than 65 years.^12^ Overall median survival for breast and prostate cancer was not reached, which might be explained by the short follow-up, slow tumor progression, and mainly by the higher proportion of patients diagnosed with early stage disease. Indeed, the American Cancer Society^7^ reports that prostate and breast cancer account for 43 and 41%, of all cancer survivors,^7,18^ with 2-year survival rates of 99 and 94.4%, respectively. As to median overall survival for stage IV patients, we found results comparable to historical data for the colon, reporting 21.5 to 23.5 months,^19-21^ and inferior outcomes for lung cancer, that reported overall survival ranged from 12 to 18.6 months.^22,23^ As we do not have data about comorbidities or the oncologic treatment, it is difficult to explain this lower overall survival for lung cancer patients as compared to the literature. Unfortunately, we could not find specific mortality data for the elderly Brazilian population with cancer.

Some limitations of our study must be acknowledged. In addition to the issues inherent to retrospective and registry studies, we had no data on specific cancer treatments, as well as no functional assessment of the patient’s health at the time of diagnosis. As far as we know, this is one of the first studies to evaluate the demographic profile of elderly patients diagnosed with solid tumors in Brazil, and should be helpful in guiding the development of health policies for this population.

## CONCLUSION

In conclusion, it appears that older adults have similar epidemiological profiles and outcomes as compared to younger individuals. Public health policies and geriatric assessment tools should be used for better coverage regarding early diagnosis and treatment.

## References

[1] National Comprehensive Cancer Network (NCCN) guidelines. Senior Adult Oncology.

[2] Brasil, Ministério da Saúde (2012). Indicadores demográficos: proporção de idosos na população.

[3] Instituto Brasileira de Geografia e Estatística (2015). População por sexo e grupos de idade 1980 – 2050.

[4] Fortin M, Hudon C, Haggerty J, Mv Akker, Almirall J (2010). Prevalence estimates of multimorbidity: a comparative study of two sources. BMC Health Serv Res.

[5] Gundrum JD, Go RS (2012). Cancer in the oldest old in the United States: current statistics and projections. J Geriatr Oncol.

[6] Brasil, Ministério da Saúde (2012). Indicadores de morbidade: taxa de incidência de neoplasias malignas.

[7] American Cancer Society (2013). Cancer Facts & Figures 2013.

[8] Guerra MR, Gallo CV, Mendonça GA (2005). Risco de câncer no Brasil: tendências e estudos epidemiológicos mais recentes. Rev Bras Cancerol.

[9] Instituto Nacional de Cancer (2008). A situação do câncer no Brasil. Ações de enfermagem para o controle do cancer: uma proposta de integração ensino-serviço.

[10] Mendonça GA (1993). Câncer na população feminina brasileira. Rev Saude Publica.

[11] Amorim VM, Barros MB, César CL, Carandina L, Goldbaum M (2006). Fatores associados à não realização do exame de Papanicolaou: um estudo de base populacional no Município de Campinas, São Paulo, Brasil. Cad Saude Publica.

[12] Surveillance, Epidemiology, and End Results Program (SEER) (2013). Turning Cancer Data Into Discovery. Cancer Statistics. Fast Stats.

[13] Walter LC, Covinsky KE (2001). Cancer screening in elderly patients: a framework for individualized decision making. JAMA.

[14] Karnakis T (2011). Oncogeriatria: uma revisão da avaliação geriátrica ampla nos pacientes com câncer. Rev Bras Med.

[15] Brasil, Ministério da Saúde (2011). Indicadores de mortalidade: mortalidade proporcional por grupo de causas.

[16] Instituto Nacional do Câncer (2013). Atlas de mortalidade por câncer. vigilância do câncer e fatores de risco.

[17] VanderWalde A, Pal SK, Reckamp KL (2011). Management of non-small-cell lung cancer in the older adult. Maturitas.

[18] American Cancer Society (2012). Cancer Treatment and Survivorship Facts & Figures 2012-2013.

[19] Grothey A, Sargent D, Goldberg RM, Schmoll HJ (2004). Survival of patients with advanced colorectal cancer improves with the availability of fluorouracil-leucovorin, irinotecan, and oxaliplatin in the course of treatment. J Clin Oncol.

[20] Van Cutsem E, Köhne CH, Láng I, Folprecht G, Nowacki MP, Cascinu S (2011). Cetuximab plus irinotecan, fluorouracil, and leucovorin as first-line treatment for metastatic colorectal cancer: updated analysis of overall survival according to tumor KRAS and BRAF mutation status. J Clin Oncol.

[21] Hurwitz H, Fehrenbacher L, Novotny W, Cartwright T, Hainsworth J, Heim W (2004). Bevacizumab plus irinotecan, fluorouracil, and leucovorin for metastatic colorectal cancer. N Engl J Med.

[22] NSCLC Meta-Analyses Collaborative Group (2008). Chemotherapy in addition to supportive care improves survival in advanced non-small-cell lung cancer: a systematic review and meta-analysis of individual patient data from 16 randomized controlled trials. J Clin Oncol.

[23] Mok TS, Wu YL, Thongprasert S, Yang CH, Chu DT, Saijo N (2009). Gefitinib or carboplatin–paclitaxel in pulmonary adenocarcinoma. N Engl J Med.

