# Supplementary material for: Clinical features and overall survival among elderly cancer patients in a tertiary cancer center
Source: Einstein (Sao Paulo). 2015 Oct-Dec;13(4):487–91. doi: 10.1590/S1679-45082015AO3067 (PMC4878619; doi:10.1590/S1679-45082015AO3067)
Supplement: Translation

# Translation: Características clínicas e de sobrevida global em pacientes oncológicos idosos num centro oncológico terciário

**Authors:** Yuri Philippe Pimentel Vieira Antunes, Diogo Diniz Gomes Bugano, Auro del Giglio, Rafael Aliosha Kaliks, Theodora Karnakis, Lucíola de Barros Pontes

**Affiliations:** 1 Hospital Israelita Albert Einstein, São Paulo, SP, Brasil.; 2 Hospital do Coração, São Paulo, SP, Brasil.

**Keywords:** Neoplasias/epidemiologia, Neoplasias/mortalidade, Oncologia, Idoso

## Abstract

**Objetivo:**

Avaliar o perfil epidemiológico e a sobrevida global em uma grande população de indivíduos idosos diagnosticados com tumores sólidos, em um hospital terciário.

**Métodos:**

Estudo retrospectivo que incluiu pacientes com idade >65 anos, diagnosticados com tumores sólidos entre janeiro de 2007 e dezembro de 2011, no Hospital Israelita Albert Einstein, São Paulo, Brasil. Os prontuários médicos foram revisados para se obterem informações sobre variáveis clínicas e sobrevida global.

**Resultados:**

Foi identificado um total de 806 pacientes, sendo 58,4% deles do sexo masculino. A média de idade foi de 74 anos (65 a 99 anos). Os tipos mais comuns de câncer foram de próstata (22%), colorretal (21%), de mama (19%) e de pulmão (13%), seguidos pelos de bexiga (8%), pâncreas (6%) e outros tipos (11%). A maioria dos pacientes foi diagnosticada em estágios iniciais. Depois de um seguimento médio de 27 meses (15 a 45 meses), 29% (234/806) estavam mortos, predominantemente no grupo com idade >70 anos. Para toda a coorte, a mediana de taxa de sobrevida em 2 anos foi 71%. A mediana de sobrevida global não foi alcançada dentro do período de estudo. Em análise multivariada, idade (HR: 1,35; IC95%: 1,25-1,45; p<0,001) e estadiamento (HR: 1,93; IC95%: 1,75-2,14; p<0,001) foram preditores negativos independentes de pior sobrevida.

**Conclusão:**

Os tumores mais prevalentes foram de próstata, colorretal, mama e pulmão, com uma grande proporção diagnosticada em estádios iniciais, o que reflete em um grande número de pacientes vivos até o último seguimento.

## INTRODUÇÃO

O câncer é uma das principais causas de morte na população idosa. Mais de 50% de todos os tipos de câncer, e cerca de 70% de mortes relacionados a câncer ocorrem em indivíduos com idade de 65 anos ou mais.^1^ Atualmente no Brasil, há mais de 20 milhões de indivíduos com mais de 65 anos de vida; os dados oficiais estimam que esse número chegue perto de 39 milhões em 2040.^2,3^

Os pacientes idosos formam uma população heterogênea em termos de comorbidades, o que pode interferir no tratamento e no prognóstico. Além disso, há diferenças na prevalência de tipos específicos de câncer e nos fatores de risco em idosos em comparação aos mais jovens. Assim, o primeiro passo para uma abordagem razoável de políticas de rastreamento e saúde para os idosos é determinar as características epidemiológicas do câncer nessa faixa etária para cada país ou região.^4,5^

## OBJETIVO

Avaliar o perfil epidemiológico e a sobrevida global em uma grande população de indivíduos idosos diagnosticados com tumores sólidos, em um hospital terciário.

## MÉTODOS

Realizamos uma coorte transversal usando o registro do Hospital Israelita Albert Einstein como banco de dados. Trata-se de um hospital particular, localizado na cidade de São Paulo (SP), Brasil. Os pacientes com 65 anos ou mais, diagnosticados com tumores sólidos, e registrados entre janeiro de 2007 e dezembro de 2011, foram os sujeitos desta análise.

Os prontuários e os bancos de dados eletrônicos institucionais foram revisados para se obterem informações sobre sexo, idade ao diagnóstico, tipo de câncer e sobrevida global. Não pudemos estabelecer mortalidade específica pelo câncer nem por todas as causas para uma grande proporção de pacientes.

A sobrevida global foi definida como a duração de tempo a partir do diagnóstico até a morte, e os pacientes foram censurados em caso de perda de seguimento (coleta até 21 de março de 2013). A sobrevida global foi estimada usando os métodos de Kaplan-Meier. O impacto de cada tratamento sobre a sobrevida foi calculado por meio do modelo de regressão de Cox.

A classificação do TNM* Classification of Malignant Tumors, *sétima edição, foi utilizada para descrever e classificar todos os diferentes estádios de câncer deste artigo.

Este projeto foi aprovado pelo Comitê de Ética Institucional, CAAE: 19159813.3.0000.0071. Todas as análises foram feitas com Stata SE 10.1 (StataCorp. College Station, Texas, Estados Unidos), com um valor de alfa bilateral de 0,05, exceto quando especificado de outra forma.

## RESULTADOS

### Perfil clínico e demográfico

Entre 1º de janeiro de 2007 e 31 de dezembro de 2011, 806 pacientes foram identificados, sendo 58,4% do sexo masculino. A média de idade foi 74 anos (65 a 99 anos), distribuída por faixas etárias: 65 a 69 anos (32%); 70 a 79 anos (45%); ≥80 anos (23%).

Os tipos mais comuns de câncer foram de próstata (22%), colorretal (21%), de mama (19%), e de pulmão (13%), seguidos pelos de bexiga (8%), pâncreas (6%) e outros tipos (11%). Os tipos menos comuns de câncer incluíram os do sistema nervoso central, tireoide, duodeno, estômago e esôfago, e a maioria dos pacientes foi diagnosticada em fase inicial (Tabela 1). A única exceção foi naqueles casos de câncer de pulmão, em que 43% foram diagnosticados com doença avançada.

**Tabela 1:** Distribuição por estádio dos tipos mais prevalentes de câncer em 806 pacientes idosos com diagnóstico de tumores sólidos no Hospital Israelita Albert Einstein (2007-2011)

Tipo de câncer	n (%)	Estádio do câncer n (%)					Não relatado n (%)
		0	1	2	3	4	
Próstata	175 (100)	-	4 (2,3)	137 (78,3)	21 (12)	13 (7,4)	-
Colorretal	171 (100)	9 (5,2)	46 (26,9)	51 (29,9)	38 (22,2)	25 (14,6)	2 (1,2)
Mama	152 (100)	19 (12)	65 (43)	39 (25,8)	22 (14,6)	7 (4,6)	-
Pulmão	106 (100)	-	28 (26,4)	10 (9,4)	21 (19,8)	46 (43,4)	1 (1)
Bexiga	67 (100)	33 (49)	18 (27)	5 (8)	-	11 (16)	-
Pâncreas	46 (100)	4 (8,7)	5 (10,9)	13 (28,2)	3 (6,5)	20 (43,5)	1 (2,2)
Gástrico	34 (100)	-	5 (14,7)	4 (11,8)	7 (20.6)	14 (41,1)	4 (11,8)
Tireoide	22 (100)	-	10 (46)	2 (9)	6 (27)	4 (18)	-

*A descrição de estádio de câncer para os cânceres menos frequentes não é mostrada aqui. Tumores do sistema nervoso central não são estadeados usando a classificação TNM.

### Sobrevida global

Após um seguimento mediano de 27 meses (15 a 45 meses), 29% dos pacientes (234/806) faleceram, predominantemente no grupo com mais de 70 anos (Tabela 2). Para a coorte toda, a mediana da taxa de sobrevida de 2 anos foi de 71% (Figura 1). As taxas de sobrevida foram piores nos pacientes com câncer de pulmão (Figura 2). A mediana de sobrevida global não foi alcançada dentro do período do estudo.

**Tabela 2:** Distribuição por faixa etária e tipo de câncer em 234 mortes de pacientes idosos em um centro oncológico em São Paulo, Brasil

	n (%)
Mortes	
Sim	234 (29,1)
Não	572 (70,9)
Distribuição de idade de pacientes que morreram (anos)	234
65-69	54 (23)
70-79	88 (37,6)
≥80	92 (39,3)
Tipos de câncer	234
Pulmões	69 (29,5)
Colorretal	49 (21)
Pancreático	30 (12,8)
Bexiga	24 (10,2)
Sistema nervoso central	16 (6,8)
Mama	15 (6,4)
Próstata	13 (5,5)
Outros (câncer gástrico, esofágico, e de tireoide)*	18 (7,7)

* Carcinoma gástrico, esofágico e tireoidiano.

**Figura 1:** Sobrevida global específica para idade em pacientes idosos com diagnóstico de tumor sólido, no Hospital Israelita Albert Einstein, São Paulo, Brasil (2007-2011)

**Figura 2:** Sobrevida global de pacientes diagnosticados com câncer de próstata (A), mama (B), colorretal (C) e de pulmão (D), conforme estádio, em idosos com tumores sólidos, no Hospital Israelita Albert Einstein, São Paulo, Brasil (2007-2011)

Para pacientes com câncer de mama e de próstata, em função da curta mediana de seguimento, não há atualmente dados maduros disponíveis de sobrevida global; entretanto, a taxa de sobrevida de 2 anos foi 90 e 92,6%, respectivamente. Com relação aos pacientes com câncer colorretal e de pulmão, a mediana de sobrevida global foi 28,5 meses e 15 meses para todos os estágios, respectivamente. Considerando apenas pacientes com doença de estádio IV, a mediana de sobrevida global foi 24 meses para o câncer colorretal, e 7 meses para câncer de pulmão. Em uma análise multivariada usando um modelo que inclui tipo, idade e estádio de doença no diagnóstico inicial, a idade (HR: 1,35; intervalo de confiança de 95% − IC95%: 1,25-1,45; p<0,001) e estádio da doença (HR: 1,93; IC95% 1,75-2,14; p<0,001) foram preditores negativos independentes de pior sobrevida.

## DISCUSSÃO

Procuramos avaliar, neste estudo, o perfil epidemiológico de uma população de pacientes idosos diagnosticados com tumores sólidos, em um hospital particular no Brasil. Nossos dados mostraram predominância discreta de pacientes do sexo masculino (58%). Os tipos mais prevalentes de câncer foram de próstata, de mama, cólon e pulmão. Tais padrões são compatíveis com dados recentes do Instituto Nacional de Câncer José Alencar Gomes da Silva (INCA) e da *American Cancer Society *(ACS),^6,7^ que incluem esses tumores dentre os cinco tipos mais comuns para todas as faixas etárias, indicando a representatividade de nossa amostra. As únicas exceções foram a menor incidência de tumores do colo do útero e estômago, em comparação com as estatísticas para a população geral,^6,8^ mais provavelmente em função do viés de seleção da nossa população.

Nossos resultados também mostraram que os tumores foram diagnosticados em estádios mais precoces que os da média nacional,^9^ provavelmente devido às características de um centro médico particular. Supõe-se que uma grande proporção desses pacientes foi acompanhada de forma apropriada por seus médicos e deve ter sido rastreada com uma frequência maior que a da população em geral.^10,11^ Ainda, nossos achados de estadiamento concordaram com aqueles relatados pelas estatísticas da *Surveillance*, *Epidemiology, and End Results(SEER*), de 2000 a 2010,^12^ em que uma grande proporção de tumores localizados foi diagnosticada como câncer de cólon, próstata, e mama em indivíduos com mais de 65 anos. Por outro lado, para câncer de pulmão, os dados de SEER mostraram 50,6% de casos como doença estádio IV, comparável com 43,4% de nossos pacientes.^12^

A detecção precoce do câncer pode resultar em tratamentos menos agressivos e melhores desfechos.^7^ Hoje em dia, há muita controvérsia sobre o uso de testes de rastreamento de câncer em idosos, como é indicado por diferentes pontos de corte para idade, recomendados por várias diretrizes, principalmente em função do baixo número de estudos clínicos neste subgrupo.^13,14^ Para auxiliar nas decisões individuais a respeito de rastreamento de câncer nessa população heterogênea, considerando potenciais riscos e benefícios, e levando em conta a expectativa de vida, seria importante realizar uma avaliação geriátrica de cada paciente antes de tomar qualquer decisão a esse respeito.

No Brasil, 66% de todas as mortes relacionadas ao câncer entre 2007 e 2011 ocorreram em pacientes com mais de 60 anos.^15,16^ Em nossa amostra, câncer de pulmão (30%) e de cólon (21%) apresentaram as maiores taxas de mortalidade. Esse padrão diverge das tendências nacionais, em que as mamas e a próstata são os principais órgãos afetados por mortalidades pelo câncer.^15,16^ Ademais, nossos dados mostram mais similaridade com a população da América do Norte, em que o número de mortes estimado para 2013 para câncer de pulmão e cólon lidera os *rankings*, com 159.480 e 50.830 mortes, respectivamente.^7^

Até que ponto as tendências em mortalidade de nossa população de pacientes são representativas dos idosos no Brasil ainda precisa ser determinado. Destacamos dois fatores que estavam associados de modo significativo aos piores desfechos, conforme revelado pela nossa análise multivariada: idade (HR: 1,35; IC95%; 1,25-1,45) e estádio clínico no diagnóstico (HR: 1,93; IC95%: 1,75-2,14). Especificamente, pacientes com mais idade do que 80 anos responderam por 39% de todas as mortes na nossa coorte; não foi possível, entretanto, determinar se essas mortes tiveram relação com câncer ou se foram por outras causas. De fato, existem evidências que mostram que a sobrevida relativa pela idade no diagnóstico em pacientes com todos os tipos de câncer, combinados no seguimento de 2 anos, é maior em pacientes com menos de 65 anos do que naqueles com mais de 65 anos de idade (75,1% *versus* 69,7%).^12^

O câncer do pulmão é a principal causa de morte em países desenvolvidos.^17^ Nos Estados Unidos, a idade mediana no diagnóstico é 71 anos, sendo 68% diagnosticados aos 65 anos de idade ou mais.^17^ Dados epidemiológicos semelhantes são observados no câncer de cólon, em que cerca de 70% dos pacientes são diagnosticados com mais de 65 anos. Segundo a SEER, 21,7% de pacientes com câncer do pulmão com mais de 65 anos estão vivos no seguimento de 2 anos,^12^ o que não difere daqueles indivíduos com câncer pulmonar com menos de 65 anos (26,5%).^12^ Com respeito ao câncer de cólon, nossos dados também foram comparáveis aos da SEER, em que 73% dos pacientes com mais de 65 anos estavam vivos no seguimento de 2 anos, *versus* 79,7% para os com menos de 65 anos.^12^ A mediana de sobrevida global para câncer de mama e próstata não foi alcançada, o que talvez possa ser explicado pelo curto tempo de acompanhamento, progressão tumoral lenta, e principalmente pela maior proporção de pacientes diagnosticados com doença em estádio inicial. De fato, a *American Cancer Society* ^7^ relata que o câncer de próstata e o de mama respondem por 43 e 41% de todos os sobreviventes de câncer,^7,18^ com taxas de sobrevida de 2 anos de 99 e 94,4%, respectivamente. Quanto à mediana de sobrevida global para pacientes do estádio IV, encontramos resultados comparáveis a de séries históricas para o cólon, relatando 21,5 a 23,5 meses,^19-21^ e desfechos inferiores para câncer de pulmão, em que a sobrevida global relatada variou de 12 a 18,6 meses.^22,23^ Já que não temos dados sobre comorbidades ou o tratamento oncológico, é difícil explicar essa sobrevida global menor para pacientes com câncer de pulmão comparada com a literatura. Infelizmente, não conseguimos encontrar dados específicos de mortalidade para a população brasileira idosa com câncer.

Algumas limitações do nosso estudo têm de ser reconhecidas. Além das questões inerentes a estudos retrospectivos e de registros, não tivemos dados sobre tratamentos oncológicos específicos, e também nenhuma avaliação funcional da saúde do paciente na ocasião do diagnóstico. Até onde sabemos, este é um dos primeiros estudos a avaliar o perfil demográfico de pacientes idosos diagnosticados com tumores sólidos no Brasil, e deve ser útil no direcionamento do desenvolvimento de políticas de saúde para esta população.

## CONCLUSÃO

Em conclusão, parece que adultos mais idosos têm perfis epidemiológicos e desfechos similares em comparação aos mais jovens. Políticas de saúde pública e ferramentas de avaliação geriátrica devem ser usadas para uma melhor cobertura de diagnóstico e tratamentos precoces.
